# The Middle Classes in Hospital

**Published:** 1923-06

**Authors:** C. E. A. Bedwell

**Affiliations:** House Governor. King's College Hospital, Denmark Hill, S.E.5


### CORRESPONDENCE.

**[Correspondence on all subjects is invited, but we cannot be responsible for the opinions expressed by our correspondents, who must give name and address as a guarantee of good faith, but not necessarily for publication. Correspondents are reminded that conciseness greatly facilitates early insertion.]**

### The Middle Classes in Hospital.

*To the Editor of* THE HOSPITAL AND HEALTH REVIEW.

SIR,—In reference to the article in your last issue on "The Middle Classes in Hospital," I have pleasure in enclosing a copy of the last annual report of this hospital, from which you will see that special arrangements have been made on the lines laid down in your article. The position of King's College Hospital is exceptional among the London Hospitals, by standing in the midst of a large resident population, which includes those who are commonly known as "poor," and also a large number of "middle class."

In these circumstances, it has been found that the wards of which use has been made for private patients have been particularly acceptable. The patients frequently express appreciation of the facilities thus afforded for the equipment of a modern hospital to be at their disposal, and the sense of security due to the presence of a resident medical officer, which can be obtained for a moderate payment of a fixed amount. If any of your readers desire to have further information, I need hardly say that I should be most happy to supply particulars.

C. E. A. BEDWELL, House Governor.
King's College Hospital,
Denmark Hill, S.E.5.

[We have referred to this letter in our "Notes and Comments."—ED. THE HOSPITAL AND HEALTH REVIEW.]

